# Effects of an incentive spirometer versus a threshold inspiratory muscle trainer on lung functions in Parkinson’s disease patients: a randomized trial

**DOI:** 10.1038/s41598-023-29534-8

**Published:** 2023-02-13

**Authors:** Saiyed Farheen Mohammed Yusuf, Anjali Bhise, Shibili Nuhmani, Ahmad H. Alghadir, Masood Khan

**Affiliations:** 1 Government Physiotherapy College, Government Spine Institute, Civil Hospital, Ahmedabad, Gujarat India; 2 Department of Physical Therapy, College of Applied Medical Sciences, Imam Abdulrahman Bin Faisal University, Dammam, Saudi Arabia; 3 Rehabilitation Research Chair, Department of Rehabilitation Sciences, College of Applied Medical Sciences, King Saud University, Riyadh, Saudi Arabia

**Keywords:** Rehabilitation, Respiratory signs and symptoms

## Abstract

Upper airway obstruction, reduced maximal expiratory and inspiratory flows, reduced lung volumes, abnormal ventilatory control, and diaphragmatic dyskinesias are reported in patients with Parkinson’s disease (PD). Inspiratory muscle training (IMT) has been reported to be effective in improving respiratory functions; however, no studies have compared the effects of the incentive spirometer (IS) with the threshold inspiratory muscle trainer (TIMT) in patients with PD. The study aimed to compare the effects of IS and TIMT on maximum inspiratory pressure (MIP), 6-min walk distance (6-MWD), forced vital capacity (FVC), forced expiratory volume in 1 s (FEV1), and peak expiratory flow rate (PEFR) in patients with stage 1–3 according to the Hoehn and Yahr scale. 18 patients were randomly assigned to two groups, i.e., incentive spirometer (IS) and threshold inspiratory muscle trainer (TIMT) group. The IS group received IMT with volume-based IS, and the TIMT group received IMT with TIMT. MIP, 6-MWD, FVC, FEV1, and PEFR were measured before and after six weeks of training. In IS group: A significant increase (*p* < 0.05) was observed in MIP and 6-MWD by 18.13 and 5%, respectively. In the TIMT group: A significant increase (*p* < 0.05) was observed in MIP and 6-MWD by 30.15 and 8.94%, respectively. Both groups observed no significant difference (*p* > 0.05) in FVC, FEV1, and PEFR. When the two groups were compared, a greater increase (*p* < 0.05) was observed in the MIP and 6-MWD in the TIMT group compared to IS group. IMT with IS or TIMT for six weeks effectively increased MIP and 6-MWD in patients with stage 1–3 (Hoehn and Yahr scale) of PD. No improvement was observed in FVC, FEV1, or PEFR with any of the techniques. TIMT is more effective than IS in improving MIP and 6-MWD.

## Introduction

Parkinson's disease (PD) is a neurodegenerative disorder that is progressive and characterized by muscle rigidity, tremors, postural instability, bradykinesia, and autonomic dysfunctions^1,2^. It is a common movement disorder in the world^2^. In this disorder, muscle strength is generally not reduced; however, the functioning of repetitive peripheral movements is affected. Therefore, these patients are able to perform single motor activities; however, repetitive and complex movements are performed with difficulty^2^. These motor disorders in patients with PD are not only limited to muscles of extremities, but the respiratory muscles of the neck, upper airway, and rib cage are also affected^1^. Several respiratory dysfunctions have been reported to be present in patients with PD, such as upper airway obstruction, decreased maximal expiratory and inspiratory flows, reduced respiratory muscle strength, decreased lung volumes due to the presence of kyphoscoliosis, the rigidity of the chest wall, abnormal ventilatory control, pleuropulmonary complications due to medications, and diaphragmatic dyskinesias^1,3^. In one study, patients with PD were shown to have a higher perception of dyspnea than normal individuals^4^. It is reported that in the flow-volume curve of patients with PD, peak expiratory flow rate (PEFR) is the most affected variable^5^. It is postulated that decreased PEFR values in PD patients may reflect severe muscular disorder^6^.

Therefore, treating respiratory problems in PD should be part of disease management to avoid complications in the respiratory system. However, no fixed respiratory protocols are suggested for respiratory problems as an intervention in patients with PD. According to a systematic review by Rodríguez et al.^7^, respiratory muscle training may be a useful tool to incorporate in PD rehabilitation programs, given the statistical improvements in maximal inspiratory and expiratory pressures, swallowing function, and phonatory measurements observed following its use. Another systematic review has reported a few respiratory pieces of training to positively affect respiratory dysfunctions in patients with PD. These trainings included incentive spirometry, expiratory, and inspiratory muscle strength training^8^. These respiratory muscle training programs have been applied to improve respiratory functions in patients with neurodegenerative disorders, including PD^3,9–11^. Other studies have used inspiratory muscle training (IMT) in patients with PD and reported improvement in inspiratory muscle endurance and strength^3^. IMT promotes deep breathing, which exercises and stretches the lungs, facilitates the movement of secretions, and helps open spaces that may have narrowed or collapsed^12^. Although a systematic review by Reyes et al.^13^ concluded that to determine if IMT or expiratory muscle training is useful in enhancing respiratory function in individuals with CNS neurodegenerative disorders, the quantity and number of studies are insufficient.

A few available types of equipment can be used as part of respiratory therapy that can improve ventilation and target lung expansion in patients with PD, thus preventing and treating respiratory complications. A volume-based incentive spirometer (IS) is a mechanical breathing device used in patients with respiratory dysfunction to aid in lung expansion^14^. The IS is widely used in respiratory, physical, or speech therapy because it provides visual feedback and encourages patients to exert slow and deep inspiration^15^. When a patient breathes through this device, the volume of air inhaled into the lungs is measured, which provides visual feedback to patients. Patients are encouraged to increase this volume. Since this device is volume or flow driven, it helps prevent lung atelectasis and restore lung volumes through slow and sustained deep breathing^16^. This device is easy to use, inexpensive, easy to train, and without side effects. The IS is used for IMT to maintain or increase the inspiratory lung volume, improve sputum expectoration, and prevent lung infection after surgery^17^.

Another commonly used device for IMT is the threshold inspiratory muscle trainer (TIMT)^18,19^. This device has been used to increase exercise capacity in post-bariatric surgery^20^, post-operative coronary bypass surgery^21^, weaning from mechanical ventilation^22^, and COPD patients^19^. It is a small handheld device with a mouthpiece and a calibrated spring-loaded valve. This device has a pressure of 9–41 cm of water, and here valve controls a constant inspiratory pressure training load. Patients must inhale air under pressure so the inspiratory valve opens and air can enter the lungs. This valve can be adjusted according to the desired needs^23,24^.

Both devices (IS and TIMT) are used for IMT and use different mechanisms for their functioning. IS is a volume-based loading device, and TIMT is a pressure-based loading device; therefore, their effects may differ. Choosing the best from them will be an advantage for PD patients. No study has compared the effects of IS with the effects of TIMT when used for IMT in patients with PD. Therefore, the study aimed to compare the effectiveness of IS versus TIMT on maximum inspiratory pressure (MIP), 6-min walk distance (6-MWD), and pulmonary functions [forced vital capacity (FVC), forced expiratory volume in 1 s (FEV1), and peak expiratory flow rate (PEFR)] in patients with PD. We hypothesized that there is a significant difference between the effects of IS and the effects of TIMT on MIP, 6-MWD, FVC, FEV1, and PEFR in patients with PD.

## Methods

### Study design

Two arms pretest–posttest experimental research design was used with random allocation of participants into two groups, i.e., the Incentive spirometer (IS) group, and the threshold inspiratory muscle trainer (TIMT) group.

### Participants

**Figure 1:**
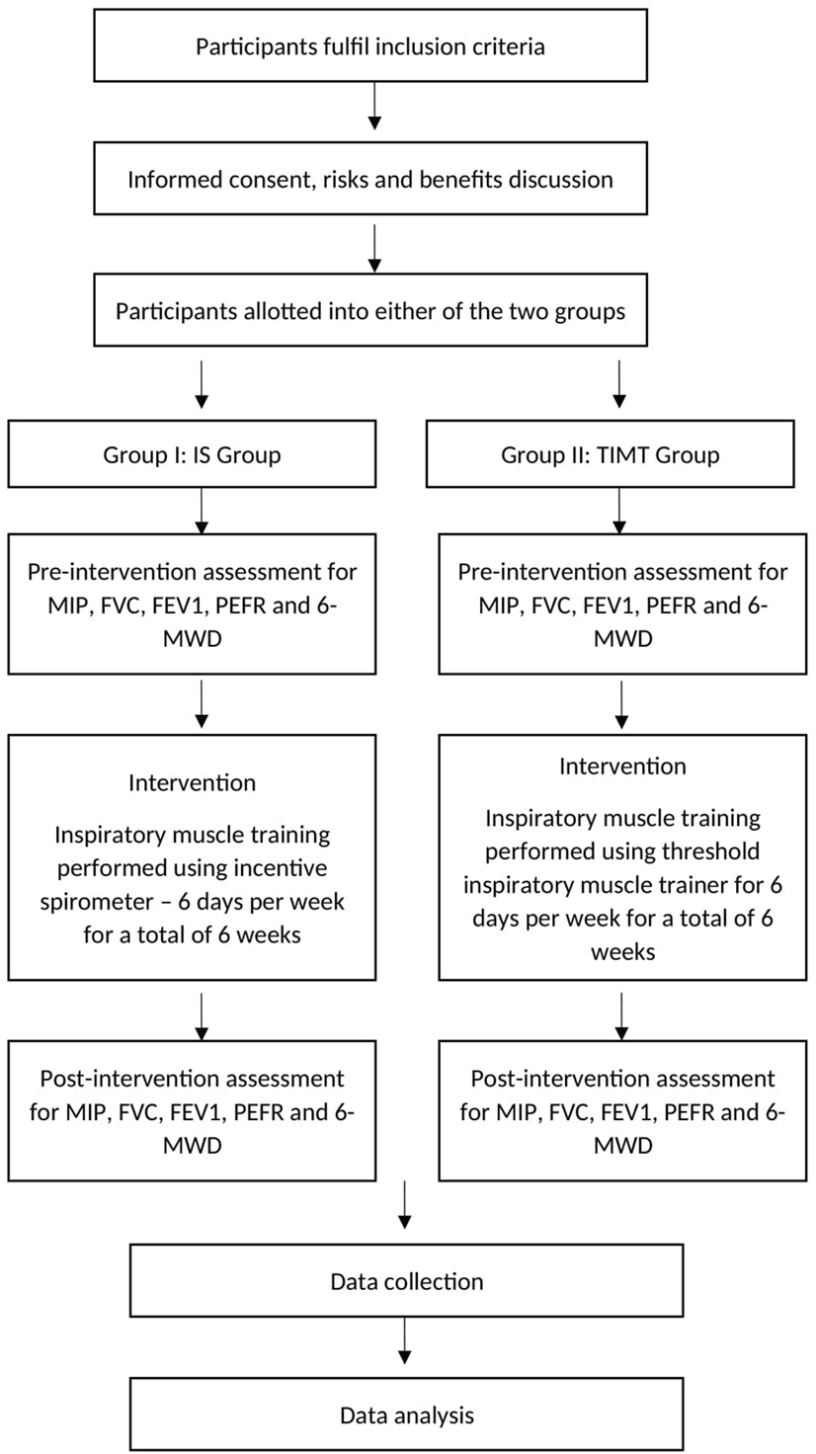
Consolidated Standards of Reporting Trials (CONSORT) flow chart showing enrollment, allocation, follow-up, and data analysis of participants.

Due to the unavailability of enough patients with PD, convenient sampling was carried out. Based on the study’s inclusion and exclusion criteria, 18 participants were recruited into the study (Table 1) (Fig. 1). Retrospectively sample size was calculated using the G*Power software, version 3.1.9.4 (Heinrich-Heine-Universitat Dusseldorf, Germany; https://www.gpower.hhu.de/) (MIP effect size (cohen’s d) = 3.028, α = 0.05, Power (1 − β) = 0.80), which revealed required total sample size as 8. Therefore, this study was sufficiently powered. Random allocation was performed using the lottery method and the website randomization.com to divide the participants into two groups (9 in each group). The examiner performed the randomization and allocation of participants to groups. The outcome assessor was unaware of the random sequence and allocation of the participants. Patients diagnosed with PD for more than five years by a neurologist in B. J. Medical College & Civil Hospital were recruited for the study. Selected participants aged 65–80, had Hoehn and Yahr scale 1–3 stage and could comprehend the commands. Patients with cardiovascular disorders, a history of smoking, psychological impairment, insufficient verbal/intellectual understanding, dementia, unstable vital parameters, and those unable to perform pulmonary function tests due to anatomical abnormalities or severe respiratory pathology were excluded from the study. Ethical approval was obtained from the institutional ethics committee of B. J. Medical College & Civil Hospital (File id: GSITESC/24/16). The study was conducted at B. J. Medical College & Civil Hospital from February 2017 to May 2018. The study conformed to ‘The Code of Ethics of the World Medical Association (Declaration of Helsinki)’. This study had been registered retrospectively in a registry of clinical trials (clinicaltrial.gov, ID: NCT05201742; date of registration: 21/01/2022). Before initiating any intervention, the risks and benefits of participation were discussed with all participants, who agreed voluntarily and signed the informed consent form.

**Table 1:** Demographic and dependent variables data, n = 9 each group, *p* values for Shapiro–Wilk test of normality for baseline values of dependent variables and *p* values for independent t-test for comparison of demographic and baseline values of dependent variables.

	IS group		TIMT group		Independent t-test *p* values
	Mean ± SD	Shapiro–Wilk *p* values	Mean ± SD	Shapiro–Wilk *p* values	
Age (years)	70.22 ± 6.18		69.67 ± 5.89		0.84
Weight (kg)	66.89 ± 10.82		60.78 ± 11.24		0.25
Height (cm)	162.78 ± 9.08		166.11 ± 8.92		0.44
BMI (kg/m^2^)	25.07 ± 1.88		21.95 ± 3.24		0.02*
MIP_Pre (cmH_2_O)	53.33 ± 20.10	0.77	53.78 ± 23.86	0.49	0.96
MIP_Post (cmH_2_O)	63.00 ± 20.77		70.00 ± 23.51		
6-MWD_Pre (m)	264.33 ± 43.93	0.34	262.11 ± 59.26	0.80	0.92
6-MWD_Post (m)	277.56 ± 43.33		285.56 ± 58.86		
FVC_Pre (Liter)	2.29 ± 0.44	0.81	1.88 ± 0.64	0.19	0.14
FVC_Post (Liter)	2.28 ± 0.49		2.11 ± 0.69		
FEV1_Pre (Liter)	1.81 ± 0.39	0.56	1.57 ± 0.47	0.16	0.24
FEV1_Post (Liter)	1.78 ± 0.30		1.71 ± 0.53		
PEFR_Pre (Liter/sec)	4.87 ± 1.82	0.25	3.94 ± 2.00	0.02*	0.32
PEFR_Post (Liter/sec)	4.70 ± 1.72		4.35 ± 2.11		

*Significant.

*BMI* Body mass index, *MIP* Maximum inspiratory pressure, *6-MWD* 6-Minute walk distance, *FVC* Forced vital capacity, *FEV1* Forced expiratory volume in 1 s, *PEFR* Peak expiratory flow rate, *SD* Standard deviation.

### Instrumentation

- PHILIPS Respironics Threshold IMT Lung Adjustable Constant Pressure Muscle Trainer (Threshold IMT, Philips Respironics, Inc., Murrysville, PA, USA)
- PORTEX Coach 2 incentive spirometer (Smiths Medical)
- Computerized Pulmonary Function Test Machine (RMS Helios 401, India)
- Portable MicroRPM (Respiratory Pressure Meter) (ICC 0.86–0.90)^25^.

### Outcome measures

- Maximum inspiratory pressure (MIP)—was measured using a portable MicroRPM (Respiratory Pressure Meter)^25–27^.
- 6-min walk distance (6-MWD)—a 30 m-long indoor walking corridor was used for this test.
- Pulmonary function tests—performed using a computerized PFT machine (RMS Helios 401). Under PFT, forced vital capacity (FVC), forced expiratory volume in 1 s (FEV1), and peak expiratory flow rate (PEFR) were measured^26,27^.

### Study protocol

1. Pre-intervention assessment: Most data were collected after breakfast and lunch (3–5 pm).
  - Maximum inspiratory pressure (MIP): The MIP was measured using a portable MicroRPM machine. The participants were asked to sit comfortably on a chair, hold the instrument with both hands, and close their lips around the mouthpiece. A nose clip was also applied to prevent the air leak from the nose. Participants were asked to exhale as much as possible, then inhale maximally for more than one second. Before the final measurement, participants were asked to familiarize themselves with the procedure^25,28^. A total of three readings were taken with a gap of 30 s or more (depending upon patients’ condition) in between procedures, and the highest MIP value was taken for data analysis.
  - Pulmonary function tests (PFT): FVC, FEV1, and PEFR were measured. The PFTs were performed using a computerized Pulmonary Function Test Machine (RMS Helios 401). Participants were asked to sit comfortably, and a nose clip was applied. The mouthpiece was placed in the mouth, and participants were asked to close their lips around it. They were asked to inhale as much as they could and then exhale maximally till no further air could be exhaled. Before taking the final measurements, participants were asked to familiarize themselves with the procedure^28–30^. A total of 3–8 readings were taken with a gap of 30 s or more (depending upon patients’ condition) in between; the highest value was taken for data analysis^11^.
  - 6-min walk distance (6-MWD): Participants were asked not to exert themselves vigorously 2 h before the test. This test was carried out indoors, along a 30 m long flat straight corridor. This distance was marked using two cones. Before starting the test, participants were asked to rest in a chair for 10 min. Then a timer was used and set at 6 min. Instructions were given to the participants. The total distance walked by the participants in 6 min was noted for baseline measurement^31^.
2. Intervention: The participants were divided into the Incentive Spirometer (IS) and Threshold Inspiratory Muscle Trainer (TIMT) groups. In group IS and TIMT, inspiratory muscle training was performed using the volume-based IS and TIMT, respectively. In both groups, the intervention was performed for six weeks^32^.
  - Incentive spirometer (IS) group: IMT was performed using the PORTEX Coach 2 incentive spirometer in this group. The participants were allowed to sit comfortably. They were asked to hold the spirometer with their hands, exhale normally, and then put the mouthpiece into the mouth with the lips closing it tightly. They were asked to inhale as slowly and deeply as possible and raise the ball in the chamber. At full inspiration, the mouthpiece was removed, followed by 3–5 s of breath-hold and then normal exhalation. This training was performed for 15 min twice a day (a total of 30 min) for six days a week^33^. The training period lasted for a total of 6 weeks. They were asked to perform this exercise at home. To maintain adherence to the treatment, each participant was given a paper with a printed timetable on which they had to put a mark each time they performed exercises.
  - Threshold inspiratory muscle trainer (TIMT) group: IMT was performed using the Threshold IMT device in this group. Participants were asked to put the mouthpiece into the mouth and put on the nose clips. Initially, training intensity was set at 30% of MIP with progression by 5% of MIP every week. The participants had to inhale from the mouthpiece, hold their breath for 2–3 s, and then exhale. This training was also performed for 15 min twice daily (a total of 30 min) six days a week. This training session also lasted for a total of 6 weeks.
3. Post-intervention assessment: MIP, PFT (FVC, FEV_1_, PEFR), and 6-MWD were measured again after the intervention, similar to the pre-intervention assessment.

### Data analysis

Data from 18 participants, with 9 participants in each group, were analyzed. SPSS statistical software, version 26 (SPSS Inc., Chicago, IL, USA), was used to analyze data. The normal distribution of baseline values of dependent variables (MIP, FVC, FEV_1_, PEFR, and 6-MWD) was assessed using the Shapiro–Wilk test of normality, which revealed a normal distribution of all variables except the baseline value of PEFR in the TIMT group. The independent t-test was used to compare both groups' demographic and baseline values of dependent variables. Paired sample t-test and repeated-measures split-plot analysis of variance (ANOVA) were used for further with-in-group and between-group analysis, respectively, for all the variables having a normal distribution. PEFR did not show normal distribution; therefore, Wilcoxon signed-rank test was performed for with-in-group analysis, and the Mann–Whitney U test was performed for between-group analysis for this variable. The confidence interval was established at 95%, and the results were considered significant for the *p* value < 0.05. The effect size for Mann–Whitney U Test was calculated using the formula *r* = *z*/√*n*. This effect size was interpreted as *r* less than 0.3—small effect, *r* between 0.3 and 0.5—medium effect, *r* greater than 0.5—large effect^34^.

### Ethics approval and consent to participate

Ethical approval was obtained from the institutional ethics committee of B.J. Medical & College Hospital (File id: GSITESC/24/16). Before initiating any intervention, the risks and benefits of participation were discussed with all participants, who agreed voluntarily and signed the informed consent form.

## Results

The demographic characteristics and mean values of the dependent variables are presented in Table 1. The independent t-test performed to compare demographic, and baseline values of dependent variables revealed no significant differences between both groups except in BMI values (*p* = 0.024) (Table 1).

### Within-group analysis

These results are presented in Tables 2 and 4.

**Table 2:** With-in-group results for MIP, 6-MWD, FVC, and FEV1, MD ± SD, df, and *p* values.

	IS group			TIMT group		
	MD ± SD	Df	*p* value	MD ± SD	Df	*p* value
MIP_Post–MIP_Pre	9.66 ± 2.23	8	< 0.001*	16.22 ± 2.10	8	< 0.001*
6-MWD_Post–6-MWD_Pre	13.22 ± 2.10	8	< 0.001*	23.44 ± 5.15	8	< 0.001*
FVC_Post–FVC_Pre	− 0.00 ± 0.14	8	0.873	0.22 ± 0.61	8	0.308
FEV1_Post–FEV1_Pre	− 0.03 ± 0.22	8	0.685	0.14 ± 0.35	8	0.242

*Significant.

*MIP* Maximum inspiratory pressure, *6-MWD* 6-min walk distance, *FVC* Forced vital capacity, *FEV1* Forced expiratory volume in 1 s, *MD* Mean difference, *SD* Standard deviation, *df* Degree of freedom.

### IS group

Participants in this group showed a statistically significant (*p* < 0.05) increase in MIP (from 53.33 ± 20.10 to 63 ± 20.77) and 6-MWD (from 264.33 ± 43.937 to 277.56 ± 43.33) by 18.13 and 5.00%, respectively. For PFTs, i.e., FVC, FEV_1_, and PEFR, no significant difference (*p* > 0.05) was observed before and after 6-weeks of intervention.

### TIMT group

Participants in this group also showed a statistically significant increase (*p* < 0.05) in MIP (from 53.78 ± 23.86 to 70.00 ± 23.51) and 6-MWD (from 262.11 ± 59.26 to 285.56 ± 58.86) by 30.15 and 8.94%, respectively. For the PFT variables, i.e., FVC, FEV1, and PEFR, no significant difference (*p* > 0.05) was observed before and after six weeks of intervention.

### Between-group analysis

These results are presented in Tables 3 and 4.

**Table 3:** Between-group (Time*Group) results for the repeated-measures split-plot analysis of variance (ANOVA).

Variable	F	*p* value	Partial eta squared (Effect size)
MIP	40.953	< 0.001*	0.719
6-MWD	30.364	< 0.001*	0.655
FVC	1.205	0.289	0.070
FEV1	1.660	0.216	0.094

*Significant.

*MIP* Maximum inspiratory pressure, *6-MWD* 6-min walk distance, *FVC* Forced vital capacity, *FEV1* Forced expiratory volume in 1 s.

**Table 4:** Wilcoxon signed-rank test and Mann Whitney U test results for PEFR, z, *p*- and effect size (r) values.

	Wilcoxon signed-rank test		Mann–Whitney U test (Between-group comparison)	
	IS	TIMT		
	PEFR_Pre–PEFR_Post	PEFR_Pre–PEFR_Post	PEFR_Pre	PEFR_Post
z	− 0.059	− 1.955	− 1.149	− 0.486
*p* value	0.953	0.051	0.251	0.627
Effect size (*r*)			− 0.270	− 0.114

*PEFR* Peak expiratory flow rate.

A statistically significant difference (*p* < 0.05) was observed between both groups for the mean difference of MIP and 6-MWD. A greater improvement (*p* < 0.05) was observed in the TIMT group compared to the IS group for both variables. For mean differences in the PFT variables, i.e., FVC, FEV_1_, and PEFR, no statistically significant difference (*p* > 0.05) was observed between both groups. For PEFR_Post values, the effect size (r) was − 0.114.

## Discussion

The present study aimed to compare the effectiveness of IS versus TIMT, when used for IMT, on lung functions in patients with PD. The results of the present study showed that MIP and 6-MWD improved with both IS and TIMT. No significant differences were observed with any of the techniques for PFT (FVC, FEV1, and PEFR). When both groups were compared, a greater improvement was observed in the TIMT group than in the IS group. When both groups were compared, large effects were observed for the mean difference of MIP (Partial Eta Squared = 0.719) and 6-MWD (Partial Eta Squared = 0.655). Therefore, TIMT has more beneficial effects on MIP and 6-MWD than IS in PD patients.

In the literature, it is already reported that in PD, patients have decreased respiratory muscle strength and endurance^35,36^. Previous studies have reported an observed reduction in lung capacity and volume in patients with PD, probably due to reduced chest compliance due to musculoskeletal limitations in the thoracic cage and decreased respiratory muscle coordination and strength^37–39^. To improve pulmonary expansion, respiratory muscle strength, and chest mobility, chest physiotherapy is considered an important part of the treatment of PD^40–42^. IMT is a part of chest physiotherapy which can be used to treat respiratory dysfunctions in PD patients. IMT can be performed using IS or TIMT. In our study, both techniques caused a significant improvement in MIP and 6-MWD.

In the present study, baseline values of MIP for Hoehn & Yahr stages 1–3 were 53.33 cmH_2_O and 53.78 cmH_2_O in IS and TIMT groups, respectively. A previous study by Dos Santos et al.^43^ reported mean values of MIP at different stages of PD. It was 59.00 cmH_2_O, 60.95 cmH_2_O, and 48.85 cmH_2_O in Hoehn & Yahr stage 1, Hoehn & Yahr stage 2 and Hoehn & Yahr stage 3/4, respectively. In the present study, the threshold IMT device was used for IMT with 30% of MIP and progression by 5% of MIP every week. Though the participants in our study did not have known pulmonary disorders, however, due to stoop and kyphotic posture, most patients have restrictive PFT patterns during PFTs. A previous study by Inzelberg et al.^3^ used resistance of 15% of the MIP in patients with PD and increased gradually 5–10% each session. Another study by Enright et al.^44^ performed IMT with 80 and 20% of maximal effort in two groups, and both groups reported improvement in MIP compared to the control group. A study by Cader et al.^22^ performed IMT with 30% MIP, increased by 10% daily, and reported increased MIP. According to Zhuang and Jia^45^, the respiratory muscle strength training protocol varies in different studies. The participants in the present study comfortably performed IMT with 30% of their MIP; therefore, we started with this and increased the resistance by 5% of MIP every week.

No study in our knowledge has used IS and TIMT for IMT and compared their effects in patients with PD; therefore, comparing the present study's findings with other studies is difficult. However, several previous studies have reported the beneficial effects of IMT on respiratory functions. Inzelberg et al. reported improved endurance and strength of the inspiratory muscle in patients with PD after specific IMT^3^. The study by Paiva et al. reported that IMT using TIMT was more effective in improving respiratory muscle strength than IS^46^. They reported that MIP increased after 15 and 30 days of training with TIMT. Similarly, Volianitis et al. reported improved MIP and performance during exercise in female rowing athletes after 11 weeks of IMT using TIMT^47^. According to them, TIMT is an effective modality for clinical approaches and improving physical performance. In addition to these studies, several other studies also suggested the effectiveness of TIMT in improving inspiratory muscle strength^19,48^.

In the present study, no significant improvements were observed in PFTs (FVC, FEV1, and PEFR) in both groups (IS and TIMT). The possible reason for this could be exercise-type specificity. There is evidence that when the same exercise type is used for testing and training, the greatest training effects will occur, called exercise-type specificity^49^. In the present study, both devices, i.e., IS and TIMT, were used to train inspiratory muscles. Therefore, the training in both groups improved MIP, not the PFTs that required expiratory force (FVC, FEV1, and PEFR). According to a systematic review and meta-analysis by Watson et al.^50^, IMT may enhance inspiratory muscular strength but not expiratory muscle strength, whereas expiratory muscle training does the opposite. However, improvement in maximal expiratory pressure (MEP) has also been reported after IMT, as in the study of Bostanci et al.^51^. Future studies could explore the training specificity of IMT and expiratory muscle training on MIP and MEP.

Gosselink et al. reported other effects of IMT, including decreased perception of discomfort in the respiratory muscles, redistribution of blood flow to the locomotor and respiratory muscles, and delay in respiratory muscle fatigue^52^. In another study, the IMT program using TIMT improved MIP compared to a program consisting of therapeutic exercises and conventional education in asthmatic children^53^. Not only is IMT reported to have beneficial effects in patients with PD, but in a study by Kuo et al. expiratory muscle strength training is also reported to increase maximum expiratory pressure, indicating that the muscle strength of expiratory muscles increased in them^54^.

In the present study, TIMT has produced better results than IS in improving MIP and 6-MWD. Therefore, the TIMT device is a better choice for IMT than IS in patients with PD. However, if TIMT is challenging to understand and use for these patients, then the physical therapist should start with a simple device that also provides feedback to the patients, which is IS. The mechanism of TIMT is pressure-based loading, and the principle of usage is inspiration against a fixed resistance pressure, which may explain the observed differences between TIMT and IS. This process is comparable to the resistance/weight exercises used to strengthen the extremities' muscles. With the TIMT device, resistance training occurs for the inspiratory muscles, which produces several benefits, including increased muscle strength and endurance, increased oxygen intake by working muscles, and reduced muscle fatigue. All of which will increase functional fitness^55–57^. The IS device, by contrast, is a volume-based loading device. Its primary usage principle is inspiration up to the TLC level, but the inspiration is not directed against resistance. As a result, the TIMT is more successful at increasing respiratory muscle strength than the IS, which results in a smaller increase in functional fitness^58^.

In the present study, the use of IS also, for six weeks, increased MIP and 6-MWD. Some previous studies showed that inspiratory muscle strength increases after IS use due to increased recruitment of motor units^59,60^. A study by Ribeiro et al. showed that IS facilitated immediate and transient positive changes in some variables of the breathing pattern, such as the increase in minute volume and tidal volume in patients with PD^61^. In their study, IS increased lung and abdominal chest wall compartments, significantly changing the chest wall tidal volume. IS is reported to facilitate the mobilization of large pulmonary volumes and cause an increase in the intra-alveolar pressure at the end of inspiration. In this device, visual stimuli are generated that provide feedback to the patients. All these phenomena result in an increase in breathing capacity after the use of IS^62–65^. It should be noted that when patients use IS and perform breathing exercises; they have to mobilize a substantial tidal volume along with a low respiratory rate, which probably results in increased muscle strength due to the increased inhalation/exhalation ratio^66^.

In the present study, the average values of 6-MWD were 264.33 and 262.11 m in IS and TIMT groups, respectively. One of the previous studies by Falvo and Earhart^67^ reported the average 6-MWD as s 391.6 m. In the present study IMT for six weeks with TIMT or IS improved 6-MWD. Zeren et al. have presented similar results and reported that 6-MWD could increase by 14% after IMT^68^. Other studies also reported similar results with IMT in patients with COPD^69^ and heart failure^70–72^.

No improvements were observed in the present study in FVC, FEV1, and PEFR in both groups. This means both IS and TIMT failed to improve pulmonary function tests in patients with PD. According to Shei et al.^55^, this might result from the adoption of standardized IMT protocols that are applied uniformly to all study participants without taking into account individual characteristics and training requirements. They suggest one strategy that may lead to better outcomes with IMT treatment: offering a tailored, personalized strategy that matches the needs of the particular patients. However, whether this approach of individualized IMT treatment is effective or not requires further good-quality randomized controlled trials.

## Limitations

The present study is not without limitations. Since recruited participants belonged to stages 1–3 of the Hoehn and Yahr modified scale and stages 4–5 were excluded, the results of this study cannot be generalized to patients with advanced PD because often more impaired lung functions are observed in later stages of this disease. Therefore, future studies should be performed on patients in the advanced stages of the disease, and those results should be compared with patients in the early stages. Also, long-term follow-up was not performed, it may be possible that observed improvements were short-lived, and after cessation of IS or TIMT training, the respiratory functions may return to baseline level. In addition to the above limitations, the history of anti-Parkinson medications was not considered when recruiting participants. It may be possible that the current use of the medications may have contributed to improvements in patient symptoms. The present study did not perform MIP according to the ATS/ERS guidelines. Due to a lack of resources, in the present study, only MIP, FVC, FEV1, and PEFR were measured; however, the measurement of more variables like maximal voluntary ventilation (MVV), total lung capacity (TLC), residual volume (RV), closing volumes, and FEF_25–75%_ could have provided more information about the effectiveness of these two instruments in PD patients and how these instruments affect the disease process.

## Conclusions

Inspiratory muscle training with IS or TIMT for six weeks improved MIP and 6-MWD in patients with early stages of PD. TIMT was more effective than IS in this population of patients. IS and TIMT did not show improvements in lung function tests (FVC, FEV1, and PEFR).

## Data Availability

The datasets used and/or analyzed during the current study are available from the corresponding author upon reasonable request.

## Abbreviations

PD: Parkinson’s disease
IMT: Inspiratory muscle training
TIMT: Threshold inspiratory muscle trainer
IS: Incentive spirometer
MIP: Maximum inspiratory pressure
6-MWD: 6-Minute walk distance
FVC: Forced vital capacity
FEV1: Forced expiratory volume in 1 s
PEFR: Peak expiratory flow rate
PFT: Pulmonary function tests
